# A case of West syndrome and global developmental delay in a child with a heterozygous mutation in the *TBL1XR1* gene: A case report

**DOI:** 10.1097/MD.0000000000033744

**Published:** 2023-05-12

**Authors:** Xiao-Hui Wu, Shuang-Zhu Lin, Zhen-Xian Liu, Yang-Fan Qi, Wan-Qi Wang, Jia-Yi Li, Qian-Dui Chen, Lu-Lu Yang

**Affiliations:** a Quanzhou Children’s Hospital, Quanzhou, Fujian Province, China; b Diagnosis and Treatment Center for Children, Affiliated Hospital of Changchun University of Chinese Medicine, Changchun, Jilin Province, China; c Changchun University of Chinese Medicine, Changchun, Jilin Province, China; d Emergency Department, The Changchun Hospital of Traditional Chinese Medicine, Changchun, Jilin Province, China.

**Keywords:** case report, global developmental delay, TBL1XR1 gene, West syndrome

## Abstract

*TBL1XR1*, also known as *IRA1* or *TBLR1*, encodes a protein that is localized in the nucleus and is expressed in most tissues. TBL1XR1 binds to histones H_2_B and H_4_ in vitro and functions in nuclear receptor-mediated transcription. TBL1XR1 is also involved in the regulation of the Wnt–β-catenin signaling pathway. Mutations in the *TBL1XR1* gene impair the Wnt–β-catenin signaling pathway’s ability to recruit Wnt-responsive element chromatin, affecting brain development. Mutations in this gene cause various clinical phenotypes, including Pierpont syndrome, autism spectrum disorder, speech and motor delays, mental retardation, facial dysmorphism, hypotonia, microcephaly, and hearing impairment.

**Case Summary:**

A 5-month-old female child was admitted with “episodic limb tremors for more than 1 month.” At the time of admission, the child had recurrent episodes of limb tremors with motor retardation and a partially atypical and hypsarrhythmic video electroencephalogram. It was determined that a heterozygous mutation in the *TBL1XR1* gene caused West syndrome and global developmental delay. Recurrent episodes persisted for 6 months following oral treatment with topiramate; the addition of oral treatment with vigabatrin did not show any significant improvement, and the disease continued to recur. The child continued to have recurrent episodes of limb tremors at follow-up until 1 year and 3 months of age. Additionally, she developed poor eye contact and a poor response to name-calling.

**Conclusion:**

We report the case of a child with West syndrome and a global developmental delay caused by a heterozygous mutation in the *TBL1XR1* gene. This study adds to our understanding of the clinical phenotype of *TBL1XR1* mutations and provides a realistic and reliable basis for clinicians.

**Key points**

• Mutations in the *TBL1XR1* gene, located at 3q26.32, cause various symptoms and clinical phenotypes.
• We report the case of a 5-month-old female child with West syndrome and global developmental delay due to a heterozygous mutation in the *TBL1XR1* gene, determined by video Electroencephalography and trio whole-exome sequencing.
• The child was treated with a combination of topiramate and vigabatrin but continued to have recurrent episodes.
• Our report enriches the understanding of the clinical phenotype of *TBL1XR1* mutations and provides a realistic and reliable basis for clinicians.

## 1. Introduction

The *TBL1XR1* gene encodes transducin-beta-like-1 X-linked receptor 1, a protein found in the nucleus and expressed in nearly all tissues. TBLR1 was first cloned by Zhang et al^[1,2]^ in 2000 and was identified as TBLR1 in 2002; it contains 514 amino acids. In 2014, Tabet et al^[3]^ sequenced TBL1XR1 at 3q26.32. TBL1 and TBLR1 interact with NCOR in addition to binding to histones H_2_B and H_4_ in vitro and contribute to nuclear receptor-mediated transcription.^[4,5]^ Furthermore, mutations in the *TBL1XR1* gene, which plays a key role in the regulation of the Wnt–β-catenin signaling pathway, reduce the pathway’s ability to recruit Wnt-responsive element chromatin, affecting brain development and causing a variety of clinical phenotypes.^[3]^

## 2. Case presentation

### 2.1. Chief complaints

A 5-month-old girl was admitted to the hospital with “episodic limb tremors for more than 1 month.”

### 2.2. History of the present illness

Prior to the age of a month, the child experienced episodic limb shaking, loss of consciousness, cyanosis of the lips, fever, and vomiting without obvious triggers. These occurred during both waking and sleeping periods. When she was 5 months old, she was still unable to raise her head, turn over, or actively grasp objects with her hands. Neither of her lower limbs could support weight when placed under her armpits.

### 2.3. History of past illnesses

The child was previously healthy, and the parents denied a history of encephalitis, poisoning, or trauma.

### 2.4. Personal and family history

The child was delivered by cesarean section at term (G2P2); there was no umbilical cord around the neck at birth and no asphyxia rescue (body weight 3550 g and height 50 cm). The parents of the child were previously healthy; the mother was healthy during pregnancy, but there was a history of special medication use and exposure to radioactive substances. The child had an older sister who was in good health and had no similar clinical manifestations. A family history of febrile seizures, epilepsy, and global developmental delay existed.

### 2.5. Physical examinations

Weight: 7.6 kg, length: 65 cm and head circumference: 41 cm. Clear consciousness, an acceptable response, steady breathing, as well as no special facial features, yellow staining, rash, bleeding points on the skin and mucous membranes, coffee milk spots, and depigmentation spots were observed. The head was not deformed; the anterior fontanel was flat (about 1.0 cm × 1.0 cm); the bilateral pupils were large, equal circles (3.0 mm in diameter), and sensitive to light reflection. The pharynx was not hyperemeal, the neck was soft, and there were no obvious abnormalities in the cardiopulmonary and abdominal examinations. The vertical neck was unstable, the head could not be raised in the prone position, inability to complete elbow support, and both lower limbs could not support weight when supporting the armpits. Extremity muscle strength class V and normal muscle tone could be noted; bilateral knee tendon reflexes and Achilles tendon reflexes could be drawn symmetrically; the double Pap, Brinell, and Kirschner signs were negative.

### 2.6. Laboratory examinations

Routine blood tests, urinalysis, blood ammonia, blood lactate, blood glucose, electrolytes, renal function, cardiac enzyme profile, hepatitis B and C antibodies, hepatitis A antibodies, blood tandem mass spectrometry, urinary organic acids, blood cytomegalovirus CMV-DNA, urinary cytomegalovirus DNA, and other physicochemical tests were normal.

### 2.7. Imaging examinations

Routine color ultrasound and chest radiography of the hepatobiliary, pancreatic, and spleen, conventional electrocardiography, cardiac ultrasound, and cranial magnetic resonance imaging showed no obvious abnormalities.

### 2.8. Further diagnostic work-up

**Figure 1.**
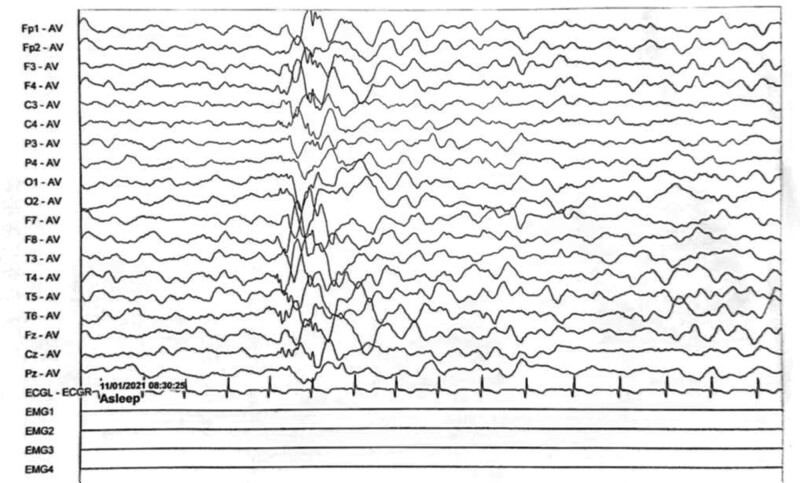
The child’s electroencephalogram (EEG) at 5 months of age. The sleep EEG shows a small number of multifocal spikes, spike-and-wave patterns, and irregular slow waves in both hemispheres. The episodic EEG shows widespread 2–4 Hz mixed slow or sharp slow waves of medium to high amplitude.

**Figure 2.**
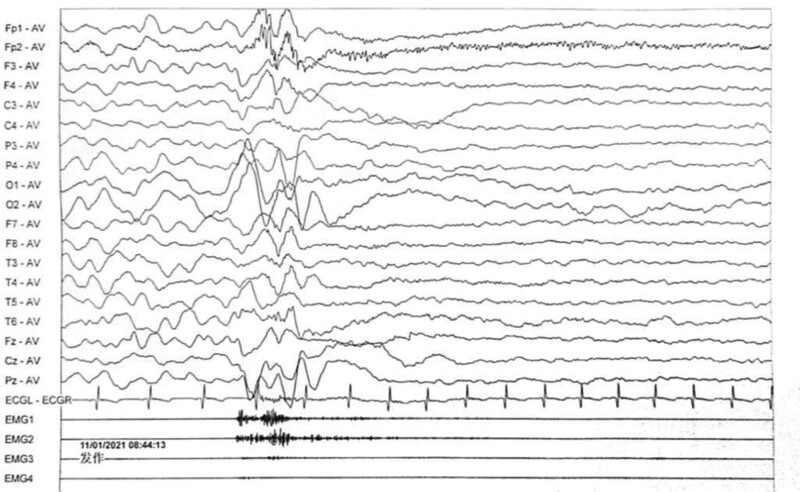
The child’s electroencephalogram (EEG) at 5 months of age. The sleep EEG shows a small number of multifocal spikes, spike-and-wave patterns, and irregular slow waves in both hemispheres. The episodic EEG shows widespread 2–4 Hz mixed slow or sharp slow waves of medium to high amplitude.

**Figure 3.**
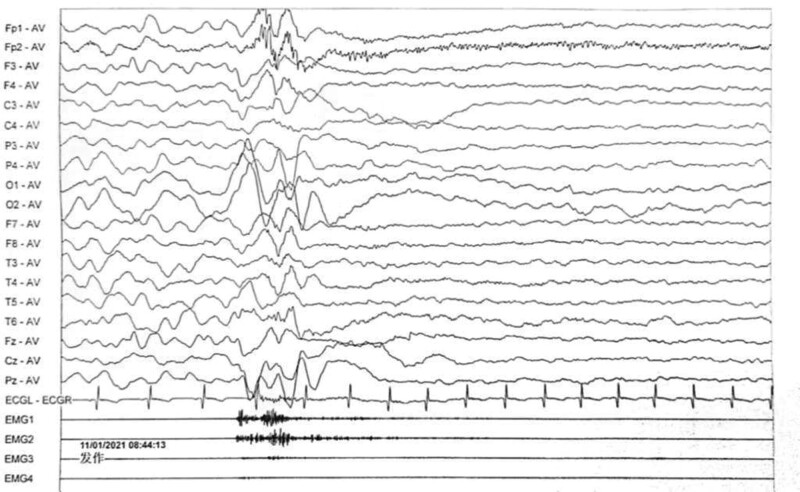
The child’s electroencephalogram (EEG) at 5 months of age. The sleep EEG shows a small number of multifocal spikes, spike-and-wave patterns, and irregular slow waves in both hemispheres. The episodic EEG shows widespread 2–4 Hz mixed slow or sharp slow waves of medium to high amplitude.

**Figure 4.**
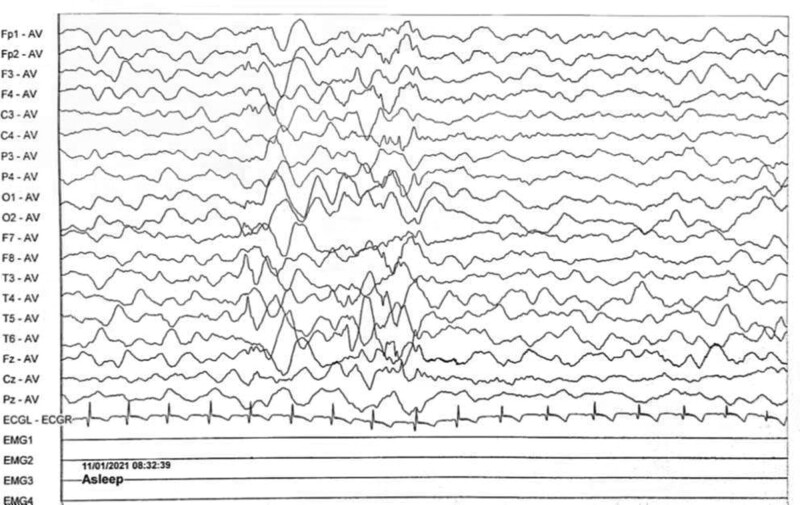
The child’s electroencephalogram (EEG) at 5 months of age. The sleep EEG shows a small number of multifocal spikes, spike-and-wave patterns, and irregular slow waves in both hemispheres. The episodic EEG shows widespread 2–4 Hz mixed slow or sharp slow waves of medium to high amplitude.

**Figure 5.**
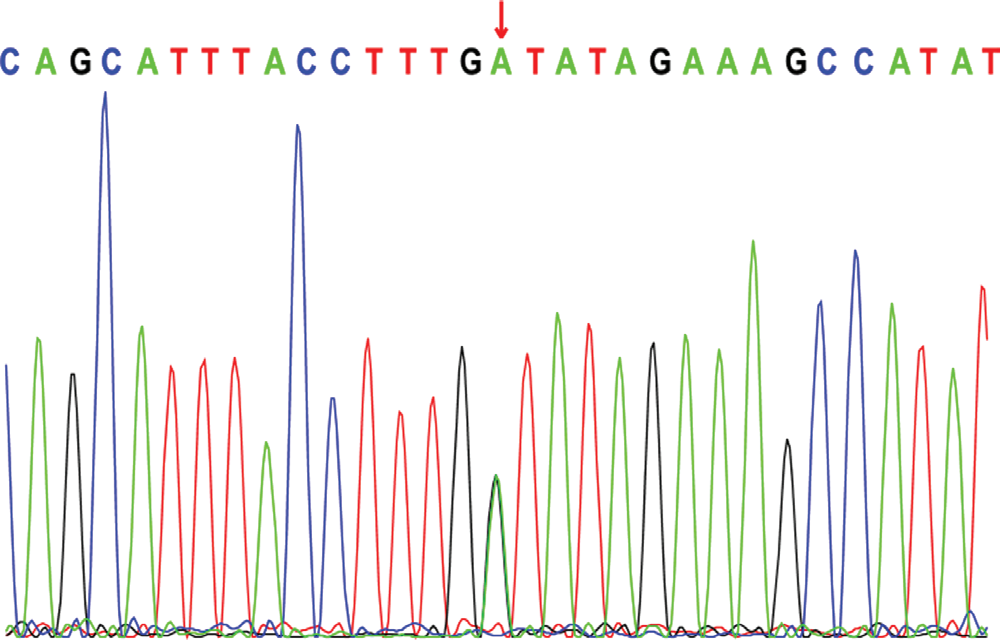
TBL1XR1: c.86 G>A (p.Gly29Asp): Proband.

**Figure 6.**
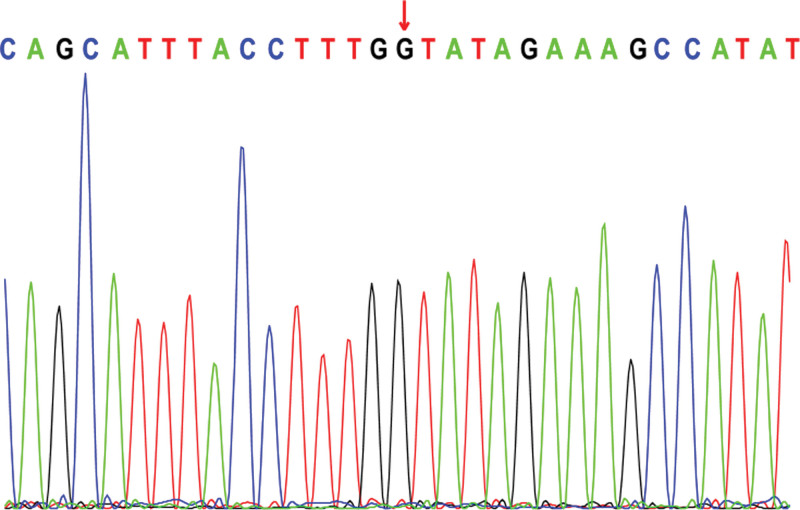
TBL1XR1: c.86 G>A (p.Gly29Asp): Father of the proband.

**Figure 7.**
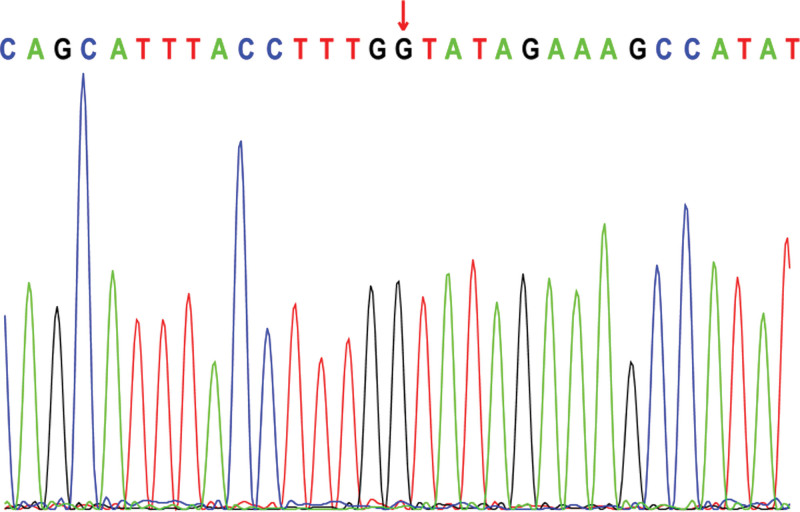
TBL1XR1: c.86 G>A (p.Gly29Asp): Mother of the proband.

Electroencephalogram: atypical hypsarrhythmic pattern (Figs. 1–4). Informed consent was obtained from the child’s parents; peripheral blood was collected from the child and parents for whole-exome sequencing. The child had a heterozygous mutation in the *TBL1XR1* gene, c.86 G>A (p.Gly29Asp), a de novo variant, and both the father and mother were wild-type (Figs. 5–7). This locus has been previously reported in the literature. The variant was considered pathogenic according to the ACMG guidelines.

## 3. Final diagnosis

The child was eventually diagnosed with West syndrome and a global developmental delay due to the *TBL1XR1* mutation.

## 4. Treatment

After admission, the child was administered vitamin B6, 100 mg/day intravenous drip, and topiramate oral antiepileptic therapy (gradually increased to 8 mg/kg/day). The child still had recurrent spasms, and undergoing vigabatrin oral therapy (gradually increased to 110 mg/kg/day) 6 months later, with recurrent seizures. Abnormal liver function, liver protection treatment, and liver function returned to normal after 6 months.

## 5. Follow-up and outcomes

In a later follow-up, we discovered that even when the child had reached the age of 1 year and 3 months, she was still unable to raise her head, actively grasp objects with her hands, sit by herself, support weight on both lower limbs while supporting the armpits, pronounce anything other than “yiya,” or make good eye contact with her parents. Her limb muscle tone was low.

## 6. Discussion

*TBL1XR1*, which encodes the protein transducin-beta-like-1 X-linked receptor 1, is located at 3q26.32. TBL1XR1 plays a critical role in the regulation of the Wnt-β-catenin signaling pathway, which has important effects on all stages of brain development. Mutations in the *TBL1XR1* gene lead to a reduction in the ability of this pathway to recruit Wnt reaction element chromatin.^[3,6,7]^ These mutations cause a variety of clinical phenotypes, including speech and motor delays, mental retardation, facial dysmorphism, hypotonia, microcephaly, hearing impairment, Pierpont syndrome, and autism spectrum disorder.^[6,8]^

TBL1XR1 can cause a number of disorders, including Pierpont syndrome, West syndrome, autism spectrum disorder, facial dysmorphism, and hypotonia.^[6,8,9]^ Pierpont syndrome is characterized by craniofacial features, hand and foot manifestations, neurodevelopmental abnormalities, and growth abnormalities such as a broad face, a high anterior hairline, narrow lid fissures, short, wide feet with plantar fat pads, deep creases and toe pads, wide “occipital” hands, deep palmar lines, and fetal pads.^[6,8,10]^ West syndrome is characterized by a triad of spasms, an arrest of psychomotor development, and hypsarrhythmia, typically manifested by sudden flexion of the trunk and neck and inversion of the arms, with onset in infancy or early childhood.^[11,12]^

The child’s episodic limb tremors and overall developmental delays at presentation drew our attention. We suspected a genetic disorder in the child after a thorough laboratory examination; therefore, we suggested a full exome test. A full exome genetic test revealed that the child had a heterozygous mutation in the *TBL1XR1* gene, c.86 G>A (p.Gly29Asp), a locus previously reported in the literature with no details regarding the associated clinical manifestations.^[13]^ She was presented with episodic limb tremors and a general developmental delay, with no obvious facial deformities or stereotyped hand movements. The child’s genotype was consistent with the clinical phenotype, and the final diagnosis of West syndrome and global developmental delay caused by a heterozygous mutation in *TBL1XR1* was confirmed.

During the follow-up period, the child displayed clinical signs, such as reduced muscle tone and poor eye contact with the parents. At the age of 1 year and 3 months, the child had poor eye contact and a poor response to name-calling, but it was not possible to determine whether this clinical presentation was due to a developmental delay or autistic tendencies, and further follow-up is required.^[14–16]^

The *TBL1XR1* mutation does not have a specific treatment, and most treatment regimens are determined by the child’s clinical phenotype. We gave the child topiramate and vigabatrin orally to manage the seizures because West syndrome and a global developmental delay were the main manifestations; however, we discovered that the seizure limb tremors continued to recur.

## 7. Conclusion

In conclusion, we report a case of West syndrome and a global developmental delay caused by a heterozygous mutation, c.86 G>A (p.Gly29Asp), in the *TBL1XR1* gene, with the main clinical manifestations being episodic limb tremors, inability to raise the head, hypotonia of the extremities, inability to actively grasp objects with both hands, and inability to sit or stand unaided. At the age of 1 year and 3 months, the child was still unable to keep her head up, sit by herself, say anything other than “yiya” and make proper eye contact. Our study has improved our understanding of the *TBL1XR1* mutation’s clinical phenotype.

## Acknowledgments

We would like to thank the patient and her family members for their contribution to this study.

## Author contributions

**Data curation:** Lulu Yang.

**Funding acquisition:** Zhen-Xian Liu.

**Investigation:** Qian-Dui Chen.

**Project administration:** Xiao-Hui Wu, Jia-Yi Li.

**Validation:** Wan-Qi Wang.

**Writing – original draft:** Yangfan Qi.

**Writing – review & editing:** Shuang-Zhu Lin.
